# Genomic Analysis of a Serotype 5* Streptococcus pneumoniae* Outbreak in British Columbia, Canada, 2005–2009

**DOI:** 10.1155/2016/5381871

**Published:** 2016-04-14

**Authors:** Ruth R. Miller, Morgan G. I. Langille, Vincent Montoya, Anamaria Crisan, Aleksandra Stefanovic, Irene Martin, Linda Hoang, David M. Patrick, Marc Romney, Gregory Tyrrell, Steven J. M. Jones, Fiona S. L. Brinkman, Patrick Tang

**Affiliations:** ^1^School of Population and Public Health, University of British Columbia, Vancouver, BC, Canada; ^2^Department of Pharmacology, Dalhousie University, Halifax, NS, Canada; ^3^Department of Molecular Biology and Biochemistry, Simon Fraser University, Burnaby, BC, Canada; ^4^British Columbia Centre for Disease Control, Vancouver, BC, Canada; ^5^Department of Pathology and Laboratory Medicine, University of British Columbia, Vancouver, BC, Canada; ^6^National Microbiology Laboratory, Public Health Agency of Canada, Winnipeg, MB, Canada; ^7^British Columbia Public Health Laboratory, Vancouver, BC, Canada; ^8^Department of Pathology and Laboratory Medicine, St. Paul's Hospital, Providence Health Care, Vancouver, BC, Canada; ^9^Department of Laboratory Medicine and Pathology, University of Alberta, Edmonton, AB, Canada; ^10^The Provincial Laboratory for Public Health (Microbiology), Edmonton, AB, Canada; ^11^Department of Medical Genetics, University of British Columbia, Vancouver, BC, Canada; ^12^Genome Sciences Centre, BC Cancer Agency, Vancouver, BC, Canada; ^13^Department of Pathology, Sidra Medical and Research Center, Doha, Qatar

## Abstract

*Background*.* Streptococcus pneumoniae *can cause a wide spectrum of disease, including invasive pneumococcal disease (IPD). From 2005 to 2009 an outbreak of IPD occurred in Western Canada, caused by a* S. pneumoniae* strain with multilocus sequence type (MLST) 289 and serotype 5. We sought to investigate the incidence of IPD due to this* S. pneumoniae* strain and to characterize the outbreak in British Columbia using whole-genome sequencing.* Methods*. IPD was defined according to Public Health Agency of Canada guidelines. Two isolates representing the beginning and end of the outbreak were whole-genome sequenced. The sequences were analyzed for single nucleotide variants (SNVs) and putative genomic islands.* Results*. The peak of the outbreak in British Columbia was in 2006, when 57% of invasive* S. pneumoniae* isolates were serotype 5. Comparison of two whole-genome sequenced strains showed only 10 SNVs between them. A 15.5 kb genomic island was identified in outbreak strains, allowing the design of a PCR assay to track the spread of the outbreak strain.* Discussion*. We show that the serotype 5 MLST 289 strain contains a distinguishing genomic island, which remained genetically consistent over time. Whole-genome sequencing holds great promise for real-time characterization of outbreaks in the future and may allow responses tailored to characteristics identified in the genome.

## 1. Introduction

*Streptococcus pneumoniae *is a ubiquitous bacterium that can cause a wide spectrum of disease, as well as being found in the normal flora of the respiratory tract in healthy individuals. Streptococcal disease ranges from ear and respiratory tract infections to invasive pneumococcal disease (IPD), which includes bacteremia and its complications, such as meningitis, arthritis, and endocarditis. In many countries, including Canada, IPD is a reportable disease to public health authorities. Prior to the introduction of pneumococcal conjugate vaccines, the incidence of IPD was highest amongst infants. However, since then, IPD rates from vaccine serotypes in children and adults have dropped significantly, with concomitant shifts in the ages of those affected and the circulating serotypes of* S. pneumoniae* [1]. Pneumococcal serotype is determined by the polysaccharide capsule, which itself is also a virulence factor [2].

In Vancouver, Canada, a large cluster of IPD associated with* S. pneumoniae* serotype 5 (multilocus sequence type or MLST 289) was identified in 2006 [3], following its initial emergence in Alberta in 2005 [4]. This increase in IPD resulted in a large outbreak of pneumococcal disease that spread throughout Western Canada (namely, the provinces of British Columbia, Alberta, Saskatchewan, and Manitoba) from 2005 to 2009 [3, 4]. In addition to being associated with a particular serotype-MLST profile, the outbreak was largely associated with certain demographic groups, affecting mostly young male adults (aged 16–65) of Aboriginal heritage. People affected were also more likely to be homeless and intravenous drug users (IDU) [3, 4].

Here we provide an update on the incidence of IPD associated with* S. pneumoniae* serotype 5 since 2009 in British Columbia (BC). Furthermore, we used a combination of whole-genome sequencing and PCR to genetically characterize the outbreak strain. This included the identification of a genomic island, which enabled us to track the extent to which the outbreak strain was causing IPD in BC.

## 2. Methods

### 2.1. Identification of* S. pneumoniae* Isolates Associated with IPD

All* S. pneumoniae* isolates throughout Canada fitting the definition of* S. pneumoniae* IPD are required to be reported to regional public health authorities. In British Columbia, invasive isolates of* S. pneumoniae* are forwarded to the BC Public Health Microbiology and Reference Laboratory for confirmation and initial serotyping followed by additional serotyping at the National Centre for Streptococcus [4]. For the purpose of this study, all IPD cases from BC with* S. pneumoniae* recovered in culture were included.

A confirmed case of IPD was defined as clinical illness, such as pneumonia with bacteremia, bacteremia without a known site of infection, and meningitis. Laboratory confirmation of* S. pneumoniae* was either through culture or detection of DNA from a normally sterile site (excluding the middle ear) (http://www.phac-aspc.gc.ca/publicat/ccdr-rmtc/09vol35/35s2/Pneumoco-eng.php). All isolates confirmed as* S. pneumoniae* are stored at −80°C.

### 2.2. Whole-Genome Sequencing

Two isolates were selected for whole-genome sequencing: BCSP1 and BCSP2. They were isolated in August 2006 and February 2010, respectively, both from patients admitted to a tertiary care hospital in Vancouver. These isolates were chosen, since BCSP1 was thought to be the index case in BC and BCSP2 was a randomly selected strain from the tail end of the outbreak in BC.

The bacteria were cultured on sheep blood agar plates and approximately 4 mm^3^ of colonial growth was used for DNA extraction using the Qiagen DNA Mini Kit as per the manufacturer's instructions. Library preparation for BCSP1 was performed as described previously [5] and for BCSP2 was performed using the Nextera kit (Illumina, San Diego, USA) as per the manufacturer's instructions. BCSP1 was sequenced on the Illumina GAIIx (50 bp paired-end reads) while BCSP2 was sequenced on the Illumina MiSeq (250 bp paired-end reads). Sequence reads were deposited to the European Nucleotide Archive under accession number PRJEB7803 (http://www.ebi.ac.uk/ena/data/view/PRJEB7803). A glossary of genomics and bioinformatics terms is listed in Table 1.

### 2.3. Bioinformatics Methods

For single nucleotide variant (SNV) detection, we chose to perform reference based assembly, whereby the raw reads are mapped or aligned to a chosen reference sequence. This involves matching each individual read to its best place in the reference genome and results in a nucleotide-by-nucleotide comparison of every sequence compared to every position in the reference. To perform reference based assembly, the software bowtie2 v0.12.8 was used to align raw reads compared to the* Streptococcus pneumoniae* 70585 reference (NCBI accession CP000918), using software input parameters –phred33 –local –dovetail –maxins 850 to maximize the quality of the assembly. It was then necessary to convert the alignment results into SNVs compared to the reference using the software SAMtools v0.1.19 [6] with the parameter indel detection turned off. The SNVs generated using SAMtools were then filtered to ensure high confidence. The parameters for filtering were (i) minimum read depth of five with at least one read in each of the forward and reverse direction; (ii) maximum read depth not greater than the highest 2.5% of the depth distribution for every nucleotide in the sample; (iii) minimum root-mean-square read mapping quality (statistic generated by SAMtools) of 20; (iv) minimum of 75% of reads supporting the consensus call of the nucleotide present at each position; and (v) calls required to be homozygous under the diploid model assumed by SAMtools (as determined from an output statistic generated by SAMtools).

Additionally, we performed* de novo *assembly in order to identify any regions present in the outbreak strains, but not the reference sequence. Prior to assembly, the software cutadapt [7] was used to remove Illumina adapters using the parameter –a for sample BCSP2. The software program Velvet v1.2.07 [8] was then used for* de novo* assembly, in conjunction with the VelvetOptimiser v2.2.0 (http://bioinformatics.net.au/software.velvetoptimiser.shtml).

After* de novo *assembly, we identified insertions not present in the 70585 reference using the software programs progressiveMauve v2.3.1 [9] and NUCmer [10]. Insertions were investigated using all contigs >500 bp generated from* de novo *assembly of the reads unmapped after reference based assembly. Regions were considered potentially unique to one strain if they did not have a match according to the backbone output file generated by Mauve or if the output of the software NUCmer indicated that <5% of one contig was not covered or >5% of a contig had double or more coverage from contigs from the other sample. Regions were verified as unique by checking that they did not match to any regions generated from the* de novo* assemblies from the entire read set using the software BLAST, and only regions >500 bp which did not match according to BLAST were considered unique. Deletions with respect to the 70585 reference were identified using a window-based approach, whereby any region of 1 kb with >500 bp missing was considered a deletion.

### 2.4. Genomic Island Identification

Genomic islands are regions of the genome with suspected horizontal origins, in that they were likely acquired from other bacteria of the same of similar species. To identify putative genomic islands in our samples, we used the software IslandPick [11]. IslandPick is a comparative genomics approach that uses both genome alignments and BLAST to identify genomic regions that are unique to a genome when compared to a set of closely related species. IslandPick was used with a minimum island size of 4000 base pairs and with the genomes of* Streptococcus pneumoniae* D39,* S. pneumoniae* TIGR4,* S. pneumoniae* R6,* S. pneumoniae* G54,* S. pneumoniae* CGSP14, and* S. pneumoniae* Hungary. Abnormal dinucleotide and codon usage bias was tested using the software IslandViewer [12], while insertion sequences were identified using the software ISfinder [13].

### 2.5. MLST

Seventeen isolates were available for multilocus sequence typing (MLST). MLST was performed using primers and conditions described at http://pubmlst.org/spneumoniae/. Resulting sequences were edited and assembled using DNAStar, Inc. and submitted to the MLST database.

Six strains sequenced by Chewapreecha et al. [14] were also included in a comparative analysis of the genomic island. Five of these six strains were investigated because they had the same serotype and sequence type (ST) as outbreak strains in this study, and the sixth was investigated because a BLASTn search of the genomic island to the NCBI wgs database identified a match to a contig from this strain. The six strains were downloaded from the NCBI short read archive in sra format and converted to fastq using the software program SRA toolkit fastq-dump [15]. They were then assembled using identical methods to those used for the two outbreak strains. Neighbor joining trees to compare the genomic island in the strains sequenced by Chewapreecha et al. to the outbreak strains were constructed using the software MEGA 6 [16].

### 2.6. PCR Detection of the Genomic Island

Two regions of the genomic island were targeted for PCR amplification. A 923 bp product at the 5′ end of the island was amplified using BF1 5′-ATCTTTTCCGCGAATCACTG-3′ (Venter 70585 2049624-2049643) and BR1 5′-TTGTATTGGAGGACCAAGC-3′ (2050519-2050537) primers. Also a 1323 bp product was amplified targeting the 3′ end with EF1 5′-GCGCTGTAATATAGGCAAAGC-3′ (2054079-2054099) and ER1 5′-ATAAGCGTGTCCGCTATCGT-3′ (2055382-2055401) primers. Bacterial colonies were inoculated into each PCR reaction consisting of 12.5 *μ*L of HotStart Taq mastermix (Qiagen), 1.25 *μ*L of each 10 *μ*M primer, and 10 *μ*L of ddH_2_O. Reactions were incubated at 95°C for 15 minutes, followed by 30 cycles each of 94°C for 30 s,* x*°C for 30 s, and 72°C for 60 s, and finally 72°C for 10 min, where *x* equals 52.5°C for BF1/BR1 and 54.0°C for EF1/ER1 primers. Additional reactions were also carried out with Ready-To-Go PCR beads (GE Healthcare) with 0.5 *μ*L of each 10 *μ*M primer and 24 *μ*L of ddH_2_O. Reactions were carried out with the same conditions for each respective primer pair. Any samples with discrepant or negative PCR results were repeated.

## 3. Results

### 3.1. Outbreak Epidemiology

**Figure 1:**
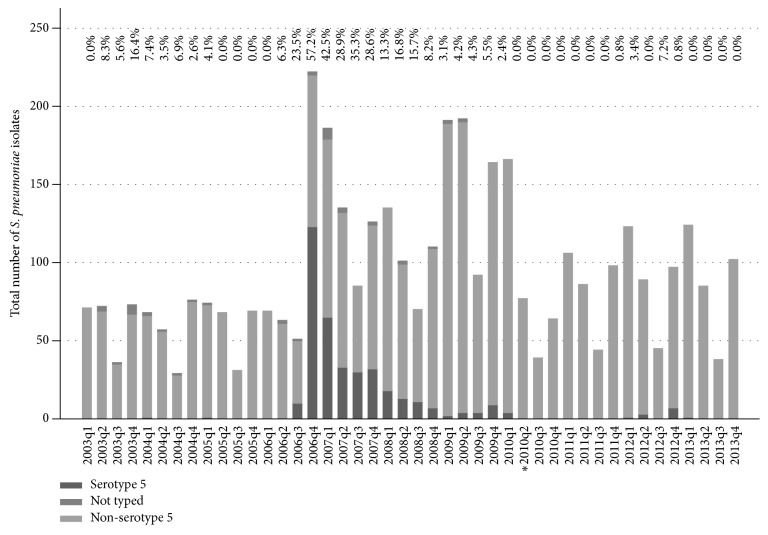
Quarterly prevalence of serotype 5 pneumococcal IPD in British Columbia. Numbers above bars are % serotype 5 isolates per quarter. *∗* is introduction of PCV13; q: quarterly (i.e., three-month period).

Investigation of* S. pneumoniae* serotypes recorded from IPD samples in BC shows that the outbreak occurred from the third quarter of 2006 to 2009 with only sporadic detections thereafter (Figure 1). During the peak, occurring in the fourth quarter of 2006, 57% of all invasive* S. pneumoniae* isolates were serotype 5. After this, the number of* S. pneumoniae* isolates that were serotype 5 continued to decrease, until June 2010, when a new multivalent pneumococcal conjugate vaccine (PCV13) was introduced in Canada, which included serotype 5.

### 3.2. Genomic Island

Genomic islands, contiguous regions of genomic sequence that have probable horizontal origins, were initially identified using IslandPick. This approach identified a 15.5 kb genomic island, which is found to be present in strains isolated from the outbreak. This island is widely present in* Streptococcus suis*; however in* S. pneumoniae*, it has only been identified in six serotype 5 isolates, the reference strain 70585 (NCBI accession CP000918), and five isolates from Maela, Thailand, in 2010 [14] (ENA accession ERR054237, ERR066355, ERR064008, ERR067846, and ERR084189). Additionally, the genomic island has been identified in a single serotype 19B ST5095 strain, also from Maela, isolated in 2008 (ENA ERR052634).

Notably, the sequence composition (i.e., proportion of each nucleotide base present) did not differ between the genomic island regions reported using IslandViewer and the rest of the genome. This suggests that the region was transferred from a genome with similar sequence composition or is an ancient transfer that has allowed amelioration of the region to the host genome.

### 3.3. PCR Based Characterization of the Genomic Island in Our Samples

From 23rd of August 2006 to 24th of July 2007, 150 serotype 5* S. pneumoniae* isolates plus 10 isolates with other serotypes, all from BC, were randomly selected for PCR testing for the outbreak-specific genomic island. Additionally, to characterize more recent isolates, one isolate was selected from the most prevalent serotypes causing invasive disease in 2013 (*N* = 38), including serotype 5 (*N* = 1). Nearly all of the serotype 5 samples were PCR positive for the genomic island (146/149, 98.0%). However, 4/149 serotype 5 samples were negative for the genomic island of which 2/14 were invasive. It should be noted that MLST and repeat serotyping were not performed on these four serotype 5 PCR-negative isolates, so we are unable to confirm that they are ST289. All other non-serotype 5 isolates were PCR-negative (Supplementary Table 1, in Supplementary Material available online at http://dx.doi.org/10.1155/2016/5381871).

### 3.4. Whole-Genome Sequencing (WGS)

97.6% of 12,160,592 reads and 97.3% of 941,010 reads for samples BCSP1 and BCSP2, respectively, were mapped to the 70585 reference, resulting in 95.6% of the reference being covered by sequence reads for both of the samples. After filtering to ensure high confidence in calls made, 90.5% and 88.7% of positions in the 70585 reference were called in BCSP1 and BCSP2, respectively.* De novo* assembly of unmapped reads produced 11 contigs >500 bp for BCSP1 with *n*50 1521, median depth 17.6, and total length 13337. For BCSP2 eight contigs >500 bp were produced with *n*50 1204, median depth 8.1, and a total length of 9475 bp.

Comparison of the two reference based assemblies revealed that they were highly similar, with only 10 variant positions between the two samples, nine of which were nonsynonymous (Tables 1 and 2). Assembly of the unmapped reads identified one region, 1476 bases in length, present in sample BCSP1 but not in BCSP2, which encoded a probable phage integrase.

Comparison of the two outbreak strains to the 70585 reference revealed 1429 SNVs where both BCSP1 and BCSP2 differ from the reference, seven genetic regions present in our samples, but not present in the reference, and 13 regions where the strains have deletions compared to the reference.

Identification of 10 SNVs between the two isolates, separated in time by 3.5 years, is suggestive of an evolutionary rate of ~1.31 × 10^−6^ substitutions per site per year, which is slightly lower than the* S. pneumoniae* evolutionary rate of 1.57 × 10^−6^ substitutions per site per year (95% CI 1.34–1.79 × 10^−6^) and 1.45–4.81 × 10^−6^ estimated by Chewapreecha et al. and Croucher et al., respectively [14, 17]. The difference of 1429 SNVs between the two outbreak samples sequenced for this study and the reference strain suggest a much more distant common ancestor.

### 3.5. Genomic Comparison of the Genomic Island between Samples

The outbreak-specific genomic island was detected in both samples from the velvet contigs generated. It differed by one variant position between the two samples in the gene producing beta-N-acetylglucosaminidase, verified from the reference based and* de novo* assembly.

**Figure 2:**
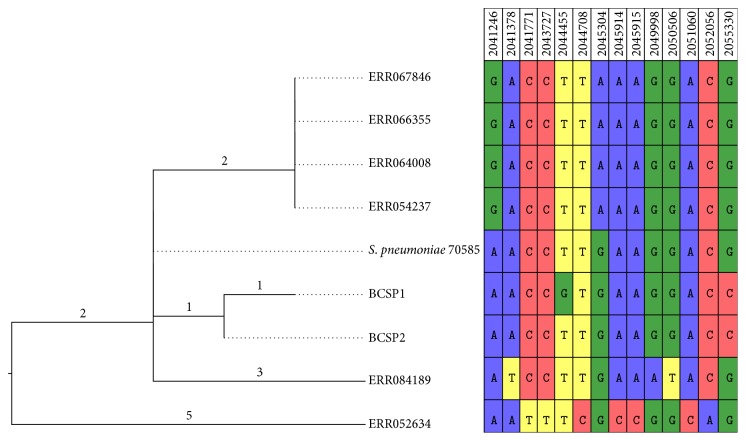
Neighbor joining tree of single nucleotide variants (SNVs) within the genomic island showing the evolutionary relationship between BCSP1 and BCSP2 (sequenced as part of this study), the 70585 reference, and six strains sequenced by Chewapreecha et al. [16] (ERR067846, ERR066355, ERR064008, ERR054237, ERR084189, and ERR052634). The table to the right shows the individual SNVs that separate the isolates, with their position in the 70585 reference at the top.

Comparison of the genomic islands identified in this study, to reference strain 70585, identified two nonsynonymous substitutions (Figure 2). The genomic islands identified from Maela were more distantly related to those in the outbreak strains. The first four serotype 5 samples from Maela, isolated from February to April 2010, were identical to one another and differed by four and three SNVs from BCSP1 and BCSP2, respectively. The genomic island from the fifth serotype 5 strain from Maela, isolated in July 2010, differed from BCSP1 and BCSP2 by five and four SNVs, respectively. These SNVs were completely different from those in the other serotype 5 Maela strains. Finally the serotype 19B strain containing the genetic island was isolated considerably earlier, in July 2008, and was more divergent from the other islands, with eight and nine SNVs from the two outbreak strains (Figure 2).

In comparison to the homologous genomic island present in* S. suis* strain TL13 [18], the* S. pneumoniae* genomic island differs by nearly 2000 variant positions of which ~350 are nonsynonymous. Furthermore, the* S. suis* island contains three large deletions compared to the* S. pneumoniae* homolog of 19, 27, and 105 nucleotides, respectively, as well as four smaller deletions of 1 (*N* = 4) or 2 (*N* = 1) bp. Of these, the 105 bp deletion spans the region between the end of the open reading frame (ORF) 2244 and the start of the subsequent ORF 2245, encoding components of the* endA* gene, and represents the insertion of the RUP unit in the pneumococcal version of the genomic island. Finally the* S. suis* genomic island also contains six insertions, of 1 (*N* = 4), 2 (*N* = 1), and 9 (*N* = 1) bp, respectively, of which the 9 bp insertion also falls in the 2245 e*ndA* gene.

## 4. Discussion

We have reported an update on the continuation of the ST289 serotype 5 pneumococcal outbreak in Western Canada from 2009 to the present date, including genetic characterization of the strain. Whole-genome sequencing of two strains representing the beginning and the end of the outbreak in BC identified a distinct 15.5 kb genetic island present in the outbreak strain, which has remained genetically consistent over time. From this sequence, a PCR assay was designed, which was able to detect the genomic island in almost all of serotype 5 strains tested from the outbreak period, demonstrating its utility as a marker for the outbreak. It is possible that the genomic island could provide an explanation for the increased severity of IPD seen during the outbreak; however, further functional experiments would be required to confirm this.

The most closely related sequence to the two strains investigated for this study is an uncharacterized strain of* S. pneumoniae *(serotype 5 and ST289), also containing the genetic island. However, five other ST289 serotype 5 strains isolated in Thailand four years after the peak of the outbreak in BC have also been demonstrated to contain the genomic island. The fact that this genomic island is present in strains from such a wide geographical range suggests worldwide spread of this* S. pneumoniae *strain. Alternatively, it could be possible that the genomic island has been present in serotype 5 pneumococci for quite some time, as suggested by the lack of sequence compositional bias between the genomic island and the rest of the genome, and the variation present within the island sequence. Interestingly the genomic island was also identified in a serotype 19B ST5059 isolate in Maela, suggesting recombination between this and a serotype 5 strain, or similar phage origins for the island region.

Comparison of BCSP1 and BCSP2, sequenced for this study, showed that, throughout the approximately four-year outbreak, the responsible strain remained genetically similar and evolved only 10 SNVs between the isolates examined, an evolutionary rate marginally slower than previous estimates. However, this rate may have been slightly greater if less stringent filtering criteria were used to identify SNVs. Despite this, the 70585 reference strain had more than 1000 SNVs compared to the two outbreak strains when the same analysis parameters were used. The conserved nature of the outbreak strain, together with its success over the outbreak period, suggests that the strain is highly adaptive to its current environment; however, further functional experiments would be required to determine which genetic features cause this. One limitation of the comparison performed between the two isolates is that they were each sequenced using a different platform (the Illumina GAIIx and Miseq). However, it has previously been demonstrated that different Illumina technologies have no impact on the conclusions drawn [19].

Genomic sequencing used in conjunction with clinical and epidemiological analysis, that is, genomic epidemiology, holds much promise in allowing rapid identification of outbreaks and notable new features associated with them [5, 20]. Furthermore, use of genomic epidemiology is likely to become increasingly routine, as the cost of whole-genome sequencing decreases and technologies for sequencing improve. As well as finding genomic islands, genomic epidemiology may identify virulence factors or antimicrobial resistance genes warranting further study [21, 22]. The identification of such features can then directly impact public health decisions; for example, it may be necessary to implement a wider vaccine campaign if a new strain harbors a genetic island with potential increased virulence and transmissibility. Furthermore, such features can also be used to design specific diagnostic assays to rapidly detect and track the spread of the outbreak strain, such as the PCR designed to identify the genomic island in this outbreak investigation. As we obtain more genome sequences and more functional genomics data, our ability to perform such analyses will only improve and become an increasingly valuable tool in infectious disease outbreak analysis and control.

## Supplementary Material

The Supplementary Material contains a summary of the PCR results for the genomic island for all the *S. pneumoniae* strains tested described in this paper.

## Figures and Tables

**Table 1:** Glossary of genomics and bioinformatics terms.

Term	Definition
Read	Short fragment of DNA sequence output by genome sequencer. Commonly 50–250 bp in length. Raw read refers to reads taken directly from the genome sequencer and in no way filtered.
Paired-end	Pairs of reads that are two ends of the same region of DNA a standard distance apart.
Depth	Number of reads mapped to a given position in the reference.
Reference based assembly	Construction of reads by mapping/aligning to a known reference sequence.
*De novo* assembly	Assembly of reads without a reference.
Adapter	Short nucleotide sequences found at the end of reads which are part of the sequencing reaction.
Insertion	Short regions of DNA present in our samples but not the reference sequence.
Contig	Contiguous regions of DNA generated by joining together raw sequence reads.
Genomic island	Regions of the genome with suspected horizontal origins, in that they were likely acquired from other bacteria of the same or similar species.
Sequence composition	Proportion of each nucleotide base present.
Codon usage bias	Regions with different sequence composition or amino acid composition compared to the rest of the genome.
*n*50	Length for which all the contigs of that length or longer contain at least half of the total of the lengths of the contigs.
Nonsynonymous	Nucleotide substitution that alters the amino acid sequence.
Open reading frame (ORF)	The part of a gene that has the potential to code for a protein.

bp: base pairs.

**Table 2:** SNVs between BCSP1 and BCSP2.

Position	70585 reference base	BCSP1 base	BCSP2 base	Qualifier	Gene product	Synonymous	Amino acid change
384518	T	A	T	SP70585_0437	Hypothetical protein	No	K → *∗*
425383	C	C	T	SP70585_0473	Helicase, RecD/TraA family	No	R → H
457503	C	C	T	*〈*Noncoding*〉*	Not applicable	—	—
568204	C	C	T	*〈*Noncoding*〉*	Not applicable	—	—
923459	T	T	G	SP70585_1004	Dihydroorotate dehydrogenase 1B	No	S → A
936681	A	A	T	SP70585_101	Competence protein	No	K → N
1389846	C	A	—	SP70585_1509	Pyridoxal biosynthesis lyase PdxS	No	E → *∗*
1675901	G	G	A	SP70585_1804	Preprotein translocase subunit SecY	No	T → I
2044455^#^	T	G	T	SP70585_2234	beta-N-Acetylglucosaminidase/beta-glucosidase (3-beta-N-acetyl-D-glucosaminidase/beta-D-glucosidase) (Nag3)	No	E → A
2104679	T	T	G	SP70585_2289	alpha-L-Fucosidase	No	S → R

^#^SNV is located in the genomic island.
